# Pelvic Electrical Neuromodulation for the Treatment of Overactive Bladder Symptoms

**DOI:** 10.1155/2011/757454

**Published:** 2011-05-14

**Authors:** Tariq F. Al-Shaiji, Mai Banakhar, Magdy M. Hassouna

**Affiliations:** Toronto Western Hospital, University Health Network, Toronto, ON, Canada M5G 2C4

## Abstract

Overactive bladder syndrome negatively affects the daily life of many people. First-line conservative treatments, such as antimuscarinics, do not always lead to sufficient improvement of the complaints and/or are often associated with disabling adverse effects leading to treatment failure. Electrical stimulation of the sacral nerves has emerged as an alternative and attractive treatment for refractory cases of bladder overactivity. Few theories attempted to explain its mechanism of action which remains elusive. It involves percutaneous posterior tibial nerve stimulation and more commonly sacral neuromodulation. For the latter, temporary sacral nerve stimulation is the first step. If the test stimulation is successful, a permanent device is implanted. The procedure is safe and reversible. It carries a durable success rate. The technique should be combined with careful followup and attentive adjustments of the stimulation parameters in order to optimize the clinical outcomes. This paper provides a review on the indications, possible mechanisms of action, surgical aspects and possible complications, and safety issues of this technique. The efficacy of the technique is also addressed.

## 1. Introduction

Overactive bladder (OAB) also referred to as the urgency-frequency syndrome, with or without urge urinary incontinence, can considerably impair the patient's quality of life. It is widely accepted that diet and life style modifications, behavioural therapy, and medication belong to the standard conservative therapeutic options and are considered as first-line measures. The International Consultation on Incontinence (ICI) guidelines states that when the first-line approach is not fully satisfactory or fails after 8–12 weeks, alternative therapies should be sought out [1]. It is worthwhile and justified to proceed to second-line therapy if patients are refractory to antimuscarinic therapy or if the treatment is contraindicated. Second-line therapies include less invasive measures such as detrusor injections with botulinum toxin (BTX) and sacral neuromodulation (SNM), whereas more invasive measures constitute surgical techniques, for example, bladder augmentation or substitution. Pelvic neuromodulation has been proven effective and is today an established treatment option for patients refractory to or intolerant of conservative treatments. It involves percutaneous posterior tibial nerve stimulation (PTNS) and more commonly SNM. This paper provides a contemporary overview of pelvic neuromodulation addressing mechanism of action, surgical and technical aspects, and safety and clinical outcomes with special emphasis on SNM.

## 2. Electrical Neuromodulation

In the settings of OAB, electrical neuromodulation devices act to modulate detrusor contractions. The use of neuromodulation is based on the knowledge that urge incontinence usually results from an imbalance of inhibitory and excitatory control systems, often causing a “hyperactive” detrusor, leading to incontinence during the filling phase [2]. In 1977, Teague and Merrill transrectally stimulated the pudendal nerve electrically in dogs which was found to activate pudendal-to-pelvic nerve reflex that depresses or eliminates uninhibited detrusor contractions [3]. Tai et al. were able to show the effectiveness of S2 sacral spinal cord microstimulation with a single electrode to induce prominent bladder and urethral sphincter responses in spinal cord-injured cats demonstrating the potential for using microstimulation techniques to modulate lower urinary tract function in patients with neurogenic voiding dysfunctions [4]. Another publication by the same group showed that in anesthetized chronic spinal cord-injured cats, impaired storage and voiding functions of the lower urinary tract could be improved by activation of the somatic afferent pathways in the pudendal nerve [5]. The authors demonstrated that electrical stimulation of the pudendal nerve at 3 Hz inhibited nonvoiding contractions during bladder filling, suppressed reflex voiding, and increased bladder capacity. In a human study, data of 22 patients with OAB, who underwent an ambulant urodynamic investigations (ACM) before and during SNM, were investigated by Scheepens et al. [6]. Blind analysis of the ACM was performed, and the detrusor activity index (DAI) was calculated as the degree of detrusor overactivity. The subjective as well as the objective results showed a decrease in bladder overactivity during SNM. During SNM, instabilities of bladder were still present; however, bladder overactivity was reduced. A significant correlation was found in DAI reduction of the ACM before and during SNM as compared to the clinical improvement on OAB symptoms.

This concept has become popular since it bridges the gap between conservative treatment and highly invasive options. Currently, these devices include SNM via surgically implanted electrodes and newer methods that deliver percutaneous stimulation of the peripheral tibial nerve. The exact mechanism of action is not well understood. A number of theories have been proposed to explain the effect of electrical neuromodulation which can be summarized as follows.

- In human subjects, it was shown that sensory input through the pudendal nerve inhibited detrusor activity and, therefore, pudendal nerve stimulation and enhancement of external sphincter tone may serve to control bladder overactivity and facilitate urine storage [7].
- The bladder tends to respond to neural stimulation initially with rapid contraction followed by slow, longer-lasting relaxation. With recurrent, repetitive stimuli produced by the electrical stimulation, there is a decay and downregulation of the bladder's response, thus reducing the detrusor muscle overactivity [8].
- Stimulation of afferent sacral nerves in either the pelvis or lower extremities increases the inhibitory stimuli to the efferent pelvic nerve and reduces detrusor contractility. One theory is that there is supraspinal inhibition of the detrusor [2]. Another assumption is that, at low bladder volumes, there is stimulation of the hypogastric nerve through activation of sympathetic fibers and at maximal bladder volume direct stimulation of the pudendal nerve nuclei in the spinal cord [9, 10].
- It is assumed that neuromodulation affects the “neuroaxis” at various levels and restores the balance between excitatory and inhibitory regulation at various locations within the peripheral and central nervous system [11].

### 2.1. Percutaneous Posterior Tibial Nerve Stimulation (PTNS)

PTNS is a minimally invasive, office-based procedure that involves percutaneous placement of a 34-gauge (ga) needle over the medial malleolus of the ankle with subchronic electrical stimulation of the posterior tibial nerve. The procedure is a 30-minute treatment session administered over a period of 12 weeks. Another method that has been described is implanting the device in the same area as well [12]. The procedure utilizes the peroneal nerve for transcutaneous access to the S3 spinal cord region. 

PTNS has shown some promise in the treatment of patients with refractory urge incontinence. McGuire et al. originally reported the first study applying PTNS in 1983 [13]. Of 22 patients with urge incontinence, 55% were cured and 32% improved. Earlier data with PTNS show excellent success rates with approximately 50% of patients showing some response with few complications noticed, albeit in low-quality studies [14]. Recently, Yoong et al. described a shortened 6-week treatment protocol with PTNS in 43 women with refractory OAB [15]. The authors showed a significant reduction in symptoms and improvement in health-related quality of life suggesting that the duration of treatment can be halved compared with the conventional 12 weeks, which would make it more acceptable and cost effective for patients. In a slightly older study from Turkey, Kabay et al. demonstrated that 12 weeks of PTNS was effective to suppress neurogenic detrusor overactivity in 19 multiple sclerosis patients [16]. Although this is a promising technology, the results of one multicenter randomized trial of 100 patients with OAB symptoms did not show a reduced rate of urinary frequency when PTNS was compared to tolterodine extended release, 4 mg daily [17]. The technique is likely to have limited applicability due to response durability since it requires regularly applying a stimulus with a percutaneous needle.

### 2.2. Sacral Neuromodulation (SNM)

SNM uses mild electrical pulses to activate or inhibit neural reflexes by continuously stimulating the sacral nerves that innervate the pelvic floor and lower urinary tract; it is also referred to as the pacemaker for the bladder. The technique was pioneered by Schmidt et al. at the University of California in San Francisco who introduced it in 1979 [18]. This was followed by further solidity by the same investigators in the mid-1980s [19, 20].**** From the first experimental use of SNM in dogs, InterStimTM therapy was developed by Medtronic (Minneapolis, Minn, USA) for use in humans. This therapy employs an implanted unilateral lead stimulating the S3 nerve root that is attached to a small pacemaker placed within a subdermal pocket in the buttock region. It is FDA approved for refractory urge incontinence, refractory urgency frequency, and idiopathic nonobstructive urinary retention. For application in OAB, the ICI level of recommendation is grade A for women and B for men [1]. The technique has been also used for conditions such as interstitial cystitis and pelvic pain syndrome. InterStim therapy has continuously evolved in terms of knowledge of its mode of action as well as in technical and surgical aspects. During the early stages of SNM, the permanent lead placement was secured by fascial fixation with the patient under general anaesthesia. However, Spinelli at al. developed a refined fixation method with twist locks or silicone anchors allowed a smaller incision under conscious sedation and, as such, a less invasive approach [21]. To further improve the technical features of the lead, Spinelli et al. designed a self-anchoring tined lead which compromises four sets of silicone tines proximal to the electrodes as an integral part of the lead body, with each tine element consisting of four flexible, pliant tines [22]. The system engages subcutaneous tissue, particularly muscle tissue, to decrease axial movement of the lead and consequent dislodgment of the stimulating electrodes. The tined lead obtained FDA approval in 2002 and opened gates for widespread application of SNM.

Preprocedure patient counselling is critical in reassuring the patient and managing treatment expectations. Once it has been decided that the patient is an appropriate candidate for InterStim therapy, implantation proceeds in 2 steps: a test phase and implantation or lead removal based on test response. The initial test phase can be performed in the office or operating room allowing for placement of the lead with a test period of 1 to 2 weeks; full implantation can be performed under local or general anesthesia. Patients are counselled that approximately 60% of patients undergoing office-based test stimulation and 70% undergoing operating room-based test stimulation will have a positive test response [23]. Response is objectively evaluated by pre- and postvoiding diaries assessing various urinary parameters. 

#### 2.2.1. One-Stage Implant

In the 1990s, Schmidt et al. devised a simple outpatient diagnostic test that involved percutaneous placement of a wire to stimulate the S3 nerve root and evaluate motor and sensory responses [24]. The innovative technique allowed for subchronic S3 nerve root stimulation, and this peripheral nerve evaluation (PNE) served as the basis for future clinical applications of SNM. In PNE, an insulated thin wire is placed into the third sacral nerve (S3) foramen in the vicinity of S3 with the patient under local anesthesia while placed on a table in the prone position. In our center, we utilize 1% plain lidocaine. The surgeon must make sure not to inject the local anesthetic into the foramen since this may lead to numbness of the underlying nerves that can preclude the desired sensory response. The sciatic notches can be palpated either uni- or bilaterally. The S3 foramen can be found one fingerbreadth off the midline at the level of the sciatic notch. The procedure is done bilaterally, and the side giving better response is chosen. Responses signalling correct placement include bellows contraction of the pelvic floor and plantar flexion of the great toe. With the in-office test stimulation, the patient will also be able to confirm correct placement with contraction or tingling of the pelvic floor muscles (e.g., rectum, vagina, scrotum, and perineum). S2 placement will demonstrate plantar flexion of the entire foot with lateral rotation, whereas S4 placement will reveal no lower extremity movement despite bellows response. Once the appropriate side and position selected, the temporary unipolar lead is connected to an external neurostimulator (external pulse generator) and taped to the skin surface. This procedure may be facilitated by the availability of office-based fluoroscopy. Response is assessed by pre- and postprocedure voiding diaries. Patients who respond favorably and demonstrate a 50% symptom improvement from baseline proceed to removal of the temporary lead followed by implantation of a quadripolar permanent lead and implantable neurostimulator placement. The leads are easily removed in the office once the test phase is complete, typically in 5 to 7 days. The duration of this test is limited to a maximum of 2 weeks because longer implantation of the temporary lead may increase the probability of bacterial contamination of the test stimulation lead [25]. Significant restrictions, such as no showering and decreased activities, also dictate short-term testing. Ideal candidates should not be obese, should have OAB without voiding dysfunction, and should not have any significant coexisting medical conditions that would make an office-based procedure difficult [23]. In addition, patients with previous sacral or coccygeal scar may not be ideal candidates since this may preclude localization and placement of the any components of the temporarily device. 

Limitations of this approach include migration of the temporary wires and a suboptimal test phase, as well as the potential discrepancy in clinical response when the permanent quadripolar lead is implanted. Short-term testing period as well as the lead migration probably explain the relatively low success rate of PNE, estimated at around 50% [26, 27]. Another observation is that to 33% of the patients who have a beneficial test stimulation with a temporary lead do not continue to have a successful outcome after the INS is implanted or, in other words, are false-positive responders [28]. Exchange of leads during the one-stage implant procedure may contribute to therapy failure during followup [29]. On the other hand, some patients who do not respond to PNE may in fact have an excellent outcome when the permanent electrode and neurostimulator/implantable pulse generator (IPG) are implanted [30]. Lead migration is considered the main factor leading to false-negative results [28].

#### 2.2.2. Two-Stage Implant

If the patient is not a candidate for office-based test stimulation or did not respond to the in-office test, test stimulation may be performed in the operating room (OR). Furthermore, the shift from PNE (one-stage implant) to a two-stage procedure helps to minimize technical-related failures and increase test efficacy and patient selection. Immediate implantation of a permanent lead aims to avoid lead migration and allows prolonged patient testing/screening [31, 32]. 

This procedure is similar to the office-based test but involves tined quadripolar leads, thus improving lead fixation and test response, and can be performed using intravenous (IV) sedation, local anaesthesia, or general anaesthesia. In case general anaesthesia is used, the anaesthetist is reminded to avoid using any long-acting muscle relaxants that may impair the ability to stimulate the sacral nerves or visualize their motor response. Fluoroscopy with C-arm should be utilized to facilitate placement. Once the right or left S3 foramen has been identified and subsequently chosen, the permanent tined lead is passed through the foramen needle. The lead is then exposed and tested in the 0, 1, 2, and 3 positions for response. Then, the sheath is carefully removed so as not to move the lead and expansion of the tines fix the lead in place. The lead is then tunnelled deeply through the subcutaneous fat to a position in the right or left buttock depending on the patient's dominant hand side where the permanent implantable pulse generator (IPG) will be placed eventually during the second stage. The lead is attached to the temporary connector and then tunneled through the subcutaneous fat to an alternative exit site. This is particularly an important step because if the patient were to get a superficial skin infection, then alternative exit site would help prevent the infection from spreading to the location of the permanent IPG and back to the lead [23]. Finally, the lead is connected to an external pulse generator and taped to the skin surface. A 7- to 14-day subchronic home test period is used to determine which patients meet criteria to have the IPG implanted. At the end of the test period, the patient returns to the OR for either removal of the lead or implantation of the IPG, depending on the subjective and objective responses. 

A prospective, randomized study showed that the two-stage implant technique of SNM has a higher success rate compared to the one-stage method, despite prior positive PNE, both in the short term and in the long term [28]. Another important study by Borawski et al. randomized 17 patients to staged implant and 13 patients to PNE [26]. The staged implant group was significantly more likely to proceed to IPG implant than the PNE group (88% versus 46%). Similar results were shown by Peters et al. who also noted that sensory response assessment at the time of implantation reduced the reoperation rate from 43% to 0% [27]. In addition, increased response rate to SNM was noted when the testing period was extended from 5 to 7 days to 14 days per implanted electrode lead [31]. The costs for the test protocol with the tined leads are much higher compared to the PNE test. Currently, the use of either one of the two screening options is arbitrary.

#### 2.2.3. Implantation

After a successful test phase, the patient is brought to the OR for implantation of the implantable generator (IPG). If the first test stimulation was office based, fluoroscopy is required to place the permanent lead. The quadripolar tined lead is inserted in a similar fashion on the side where the patient had the best in-office test response. The lead is then tunnelled deeply through the subcutaneous fat to an incision in the right or left buttock region. It is attached to the IPG and buried in the deep subcutaneous pocket. On the other hand, if the first phase was done in the OR and there is pre-existing placement of the permanent quadripolar lead, the implant stage is quick, does not require fluoroscopy, and can be performed under local or general anaesthesia. The previous incision where the temporary connector was placed in the buttock is opened, and the permanent IPG is then connected to the lead and buried in a deep subcutaneous pocket in the buttock. Buttock placement of the IPG has become an attractive alternative to subcutaneous implant in the lower part of the anterior abdominal wall because of the lower incidence of adverse events (approximately 2-fold), shorter operation time, and avoidance of patient repositioning during the operation [33]. Postoperatively, the IPG is switched on, and it is programmed with different electrodes mapping to give the patient a comfortable electrical stimulation. Patients need lifelong surveillance to manage device-related issues that may arise.

#### 2.2.4. Complications, Safety, and Clinical Results

The very nature of this mode of therapy mandates a 100% reoperation to replace the IPG at some point due to the limited longevity of the neurostimulator. Adverse events are usually related to the implant procedure and the presence of the implant or of undesirable stimulation. The most common adverse events include lead migration, implant site pain, bowel dysfunction, and infection. The majority of adverse events do not require surgical intervention. Potential lead migration can be simply resolved without significant morbidity in the majority of patients by reprogramming, reinforcing the lead, or inserting a new lead contralaterally [34]. Some patients lose benefit due to accommodation to the stimulation, but contralateral placement can be attempted to overcome this [35]. If infection is superficial, the usual management is antibiotics; however, if there is a deep infection that is not resolved with oral or IV antibiotics, then explantation of the neurostimulator is required. In case of adverse stimulation, it is commonly sufficient to change the stimulation factors (e.g., electrode mapping, pulse width, amplitude, mode, or polarity). Hijaz et al. reported a review of complication management and implant troubleshooting strategy from the Cleveland Clinic database of 214 tine lead implants [36]. One hundred and sixty-one patients (75.5%) proceeded to placement of the IPG. Seventeen patients (10.5%) had the device completely removed for infection and failure of clinical response. Twenty-six patients (16.1%) underwent device revision due to attenuation of response, infection, pain at IPG site, and lead migration. The majority of patients with revision due to poor response had abnormal impedance measurements, with equalization of impedance in 2 leads being the most common finding. As a result, the authors strongly advocate IPG interrogation with impedance testing to completely evaluate patients with response-related dysfunction.

Contraindications for the patient with an implanted device include shortwave diathermy, microwave diathermy, or therapeutic ultrasound diathermy. The diathermy's energy can be transferred through the implant and could be harmful. MRI is not recommended. Nevertheless, Elkelini and Hassouna reported on six patients with implanted sacral nerve stimulator who underwent eight MRI examinations at 1.5 Tesla conducted in areas outside the pelvis [37]. IPGs were examined before and after MRI procedures. All patients had their parameters recorded; then the IPGs were put to “nominal” status. Patients were monitored continuously during and after the procedure. During the MRI session, no patient showed symptoms that required stopping the examination. There was no change in perception of the stimulation after reprogramming of the implanted sacral nerve stimulator, according to patients' feedback. Devices were functioning properly, and no change in bladder functions was reported after MRI examinations. Another safety issue with SNM has been its effect in pregnant women and the developing fetuses. Wiseman and colleagues have addressed this issue by examining 6 eligible patients having SNM sacral who subsequently achieved pregnancy [38]. In 5 patients, the stimulator was deactivated between weeks 3 and 9 of gestation, after which 2 with a history of urinary retention had urinary tract infection. In another case, stimulation was discontinued 2 weeks before conception. The only noted complication developed in a pregnancy in which birth was premature at 34 weeks. Three patients underwent normal vaginal delivery, including 1 in whom subsequent implant reactivation did not resolve voiding dysfunction. In 3 cases, elective cesarean section was performed. All neonates were healthy. The authors concluded that when a patient on neuromodulation achieves pregnancy, the stimulation should be deactivated. If implant deactivation leads to urinary-related complications that threaten the pregnancy, reactivation should be considered. Elective cesarean section should be considered since it is possible for sacral lead damage or displacement to occur during vaginal delivery.

Several investigators have attempted to identify parameters that have predictive value in selecting the best candidates and those patients most likely to benefit from SNM therapy. Amundsen at al. reported that age >55 years and more than three chronic conditions were independent factors associated with a lower cure rate in patients implanted with a sacral neuromodulator for refractory urge incontinence [39]. They also noted that a neurologic condition may be associated with a decrease in the cure rate. Sherman et al. showed that evidence of pelvic muscle activity and test stimulation performed within 4 years were predictive factors of a positive response [40]. Other studies have demonstrated that patients with OAB symptoms and concomitant emotional disorders are far more likely to respond poorly to test stimulation, have symptom recrudescence following permanent implant, and have a higher incidence of reoperations [28, 41]. In a different study, Foster et al. showed that the reduction in 24-hour pad weight best predicted long-term patient satisfaction with SNM therapy [42]. 

There is convincing evidence for the success of SNM with the Interstim technique for refractory OAB. Several studies including RCTs and long-term observational studies reported fair clinical response between 64 and 88% of all patients [43]. All parameters investigated showed significant improvement compared to the placebo group: a 23–46% decrease in the number of voids per day, 44–77% increase in the average voided volume, 56–90% decrease in incontinence episodes per day, 64–100% decrease in pads, and 39% increase in maximum cystometric capacity [36, 41, 44–49]. Cappellano et al. showed a significant improvement in the quality of life score in patients with urgency incontinence who underwent SNM [50]. When followed up for 18 months, they were asked whether they would undergo this treatment again. 90% responded yes and 100% would recommend it to a relative or friend. Recently, Chartier-Kastler et al. published a multicenter prospective observational trial evaluating long-term effectiveness of SNM in patients with a permanent implant (2003–2009) [51]. Clinical improvement of greater than or equal to 50% was seen in 447/527 patients with OAB at 12 months followup. Clinical improvement remained relatively stable up to 60 months. Median patient satisfaction with treatment was between 60 and 80%. In another study, Leong et al. surveyed all patients who received SNM between 1990 and 2007 by mailing a questionnaire regarding satisfaction and experiences with the system [52]. Of the 275 questionnaires sent, 207 were returned for a 75% response rate. Treatment was done for OAB in 55% of the patients. Overall satisfaction with SNM was high at 90%.

Recently, several technical aspects of SNM with InterStim therapy led to the development of the InterStim II system, which received regulatory approval in Europe and the United States in 2006. InterStim II eliminates the need for extension cables and is almost 50% lighter and smaller in volume compared to the initial model. Subsequently, this allows for a smaller incision and smaller pocket to be created and thus less patient discomfort with higher patient acceptance which is of particular importance for skinny patients. However, the above-mentioned advantages come with the expense of a shorter battery life. Most new implanted IPGs are supplied with small iCon patient programmers, offering the patients the possibility to choose from up to four preset programs, provided better control of stimulation by the patient. Other available SNM technology includes the twin-chamber IPGs that can feed two electrodes providing synergetic effect.

## 3. Conclusions

Electrical neuromodulation devices act to modulate detrusor contractions. Currently, these devices include SNM and PTNS. SNM is an effective treatment modality for patients with refractory OAB and should be offered before applying more invasive, irreversible treatments. The procedure is safe and minimally invasive involving one or two-stage implantation. It carries small, treatable, and nonpermanent side effects. Although the mechanisms behind its action are still not fully understood, the therapy has been shown to be effective in the long term. Followup should include regular checks to determine efficacy of the therapy and a review of the electrical system. The SNM technology continues to evolve.
